# Comparison of Phenotypical Antimicrobial Resistance between Clinical and Non-Clinical *E. coli* Isolates from Broilers, Turkeys and Calves in Four European Countries

**DOI:** 10.3390/microorganisms9040678

**Published:** 2021-03-25

**Authors:** Octavio Mesa-Varona, Rodolphe Mader, Martina Velasova, Jean-Yves Madec, Sophie A. Granier, Agnes Perrin-Guyomard, Madelaine Norstrom, Heike Kaspar, Mirjam Grobbel, Eric Jouy, Muna F. Anjum, Bernd-Alois Tenhagen

**Affiliations:** 1 German Federal Institute for Risk Assessment (BfR), Department Biological Safety, 10589 Berlin, Germany; Mirjam.Grobbel@bfr.bund.de (M.G.); Bernd-Alois.Tenhagen@bfr.bund.de (B.-A.T.); 2 French Agency for Food, Environmental and Occupational Health and Safety (ANSES), Laboratory of Lyon, Antibiotic Resistance and Bacterial Virulence Unit, University of Lyon, 31 avenue Tony Garnier, 69007 Lyon, France; Rodolphe.Mader@anses.fr (R.M.); Jean-Yves.Madec@anses.fr (J.-Y.M.); 3 Animal and Plant Health Agency (APHA), Department of Epidemiological Sciences, Addlestone, Surrey KT15 3NB, UK; Martina.Velasova@apha.gov.uk; 4 French Agency for Food, Environmental and Occupational Health and Safety (ANSES), Fougeres Laboratory, 35133 Fougeres, France; Sophie.Granier@anses.fr (S.A.G.); Agnes.Perrin-Guyomard@anses.fr (A.P.-G.); 5 Norwegian Veterinary Institute (NVI), Department of Animal Health and Food Safety, Research Section Food Safety and Animal Health, 0454 Oslo, Norway; Madelaine.Norstrom@vetinst.no; 6 Federal Office of Consumer Protection and Food Safety (BVL), Reference Laboratories, Resistance to Antibiotics Unit Monitoring of Resistance to Antibiotics, Department Method Standardisation, 12277 Berlin, Germany; Heike.Kaspar@bvl.bund.de; 7 French Agency for Food, Environmental and Occupational Health and Safety (ANSES), Ploufragan-Plouzané-Niort Laboratory, 22440 Ploufragan, France; Eric.jouy@anses.fr; 8 Animal and Plant Health Agency (APHA), Department of Bacteriology, Addlestone, Surrey KT15 3NB, UK; Muna.Anjum@apha.gov.uk

**Keywords:** AMR, clinical isolates, non-clinical isolates, broiler, turkey, calf, *E. coli*

## Abstract

Livestock data on antimicrobial resistance (AMR) are commonly collected from bacterial populations of clinical and non-clinical isolates. In contrast to data on non-clinical isolates from livestock, data on clinical isolates are not harmonized in Europe. The Normalized Resistance Interpretation (NRI) method was applied to overcome the lack of harmonization of laboratory methods and interpretation rules between monitoring systems. Statistical analyses were performed to identify associations between the isolate type (clinical vs. non-clinical) and resistance to four antimicrobials (ampicillin, tetracycline, gentamicin, and nalidixic acid) per animal category in Germany and France. Additional statistical analyses comparing clinical and non-clinical isolates were performed with the available data on the same antimicrobial panel and animal categories from the UK and Norway. Higher resistance prevalence was found in clinical isolates compared to non-clinical isolates from calves to all antimicrobials included in Germany and France. It was also found for gentamicin in broilers from France. In contrast, in broilers and turkeys from Germany and France and in broilers from the UK, a higher resistance level to ampicillin and tetracycline in non-clinical isolates was encountered. This was also found in resistance to gentamicin in isolates from turkeys in Germany. Resistance differed within countries and across years, which was partially in line with differences in antimicrobial use patterns. Differences in AMR between clinical and non-clinical isolates of *Escherichia coli* are associated with animal category (broiler, calf, and turkey) and specific antimicrobials. The NRI method allowed comparing results of non-harmonized AMR systems and might be useful until international harmonization is achieved.

## 1. Introduction

Antimicrobials are essential to maintain the human and animal health status. They allow bacterial infections, one of the most frequent disease groups in livestock, to be controlled. However, the effectiveness of antimicrobials has been reduced due to the increase in antimicrobial resistance (AMR) caused mainly by widespread antimicrobial use (AMU) in humans and animals [1].

Global, regional, and national strategies such as the Global Action Plan (GAP) on AMR of the World Health Organization (WHO) [1], the European Union (EU) One Health Action Plan against AMR [2], and national action plans (NAP) have been implemented to limit AMR development and spread. In France, the NAPs are the “plan national de réduction des risques d’antibiorésistance en médecine vétérinaire” (Écoantibio plan) in the animal sector, the “Programme national d’actions de prévention des infections associées aux soins” (PROPIAS) in the human sector, and the “plan national de santé et d’environnement” (PNSE3) in the environment sector [3]. Germany has published the “Deutsche Antibiotika-Resistenzstrategie” (DART) [4], and the UK has published the “5-year national action plan for antimicrobial resistance 2019 to 2024” [5].

Surveillance and monitoring systems on AMR and AMU in animals, a relevant pillar of NAPs, are highly important means to (a) document the situation; (b) identify trends; (c) set up the basis for risk assessment and interventions; (d) assess effects of efforts carried out; (e) associate AMU and AMR; (f) focus and target the research [6]; and (g) advise on veterinary treatments and antimicrobial stewardship [7].

*Escherichia coli* are Gram-negative bacteria that are commonly found as commensals in the intestinal tract of humans and animals. They are also intestinal pathogens. Resistance to antimicrobials carried by *E. coli* may be spread horizontally to other bacteria [8]. Antimicrobial resistance dynamics may be assessed by monitoring resistance in commensal *E. coli*, a widely accepted AMR indicator [9,10,11,12].

Livestock data on AMR are classically collected from (a) diseased animals (clinical data) and (b) healthy animals (non-clinical data). Epidemiological cut-off values (ECOFFs) [13] and clinical breakpoints (CBPs) [14] are used to interpret antimicrobial susceptibility testing (AST) results. Whilst ECOFFs are preferred for monitoring and surveillance objectives contrasting the wild-type and non-wild type populations, CBPs define a microorganism as susceptible, susceptible-increase exposure, or resistant depending on the probability of a therapeutic treatment succeeding [10,15].

At European level, most resistance data on non-clinical isolates from food producing animals are collected according to the Commission Implementing Decision 2013/652/EU showing, therefore, a high degree of harmonization. Some European countries also have systems collecting data from diagnostic, or clinical, submissions (e.g., France, Norway, the United Kingdom, and Germany). While the European Food Safety Authority (EFSA) collects data on non-clinical isolates on the European level, limited data on clinical isolates from diagnostic submissions from livestock are collected in Europe. Currently, only the VetPath and MycoPath initiatives funded by the pharmaceutical sector in Europe publish such data [16,17,18], although there is a call to launch the European Antimicrobial Resistance Surveillance network in veterinary medicine [19].

Antimicrobial resistance data on clinical and non-clinical isolates from livestock show a lack of harmonization in various aspects within and between countries in Europe. This concerns the laboratory method (e.g., disk diffusion and microdilution), the laboratory procedure, the type of data collected (quantitative vs. qualitative data), the standards used (EUCAST, CLSI, or national standards), the interpretation criteria (ECOFFs vs. CBPs), the antimicrobial panel used, and the epidemiological data on sampled animals reported [16].

Harmonized AMU data are collected as sales data in Europe from livestock as mg/population correction unit (PCU) by the European Surveillance of Veterinary Antimicrobial Consumption (ESVAC) [16]. Antimicrobial use decreased considerably in mg/PCU from 2014 to 2017 in several countries such as Germany, France [20], and the United Kingdom [21]. In Norway, antimicrobial use was extremely low [20]. Usage data on farm level could provide a higher level of detail than sales data, addressing the association between AMU and AMR per animal and drug category more precisely [16,22,23]. Some countries have started collecting AMU at farm level [16]. However, no harmonized data collection system on AMU at farm level has been set up in Europe yet [7,16].

Studies in Estonia and Germany investigated AMR based on clinical and non-clinical isolates. The first showed on a descriptive level a higher resistance prevalence in clinical isolates in pigs and cattle [24]. However, statistical analyses carried out in the German study for broilers and turkeys showed a general pattern with lower occurrence of resistance in clinical *E. coli* isolates as compared to non-clinical [25].

To our knowledge, there is no publication statistically comparing AMR data between clinical and non-clinical isolates from national monitoring systems using different laboratory methods and procedures. Such a publication might advise political decisions to mitigate AMR in countries.

The Normalized Resistance Interpretation (NRI) method was developed to determine cut-offs statistically, using distributions of MICs or inhibition zone diameters. This procedure facilitates comparisons of AMR data from different laboratories, based on different laboratory methods and procedures [26,27].

The main objective of this work is to compare AMR data on clinical and non-clinical isolates of *E. coli* within countries in several animal categories and to describe these results across countries. The NRI method was applied to overcome the lack of harmonization in AMR regarding laboratory methods and procedures between national monitoring systems within countries.

It is plausible to expect that isolates harvested from diseased animals might carry higher levels of resistance to regular antimicrobial treatments than random isolates from healthy animals [24]. This should be studied carefully in different animal categories and countries, as it was not confirmed in the literature [25]. The hypothesis we considered in this work was that the resistance level in *E. coli* from broilers, turkeys, and calves is higher in clinical isolates than in non-clinical isolates. The counter-hypothesis is that commensals are more frequently exposed to antimicrobials, administered for other reasons, over the course of an animal’s life. We assumed that the findings on broilers and turkeys in Germany are an exception [25]. In order to challenge our hypothesis, we applied univariable and multivariable logistic regression analyses comparing resistance levels in clinical and non-clinical *E. coli* isolates within countries. The year variable was also included in the analyses. Available AMU data from countries were not included in the national statistical analyses, as AMU data showed insurmountable limitations to be analytically compared to AMR. The populations reflected in the use data were not congruent with those covered by the resistance testing (e.g., AMU data from poultry vs. resistance from broilers and turkeys and resistance data from calves vs. use data in cattle in France). However, attempts to compare AMU and AMR were performed at descriptive level.

## 2. Materials and Methods

### 2.1. Data Collection and Processing

Phenotypic AMR data of *E. coli* were collected from different sources between 2014 and 2017. Caecal samples from broilers, turkeys, fattening pigs, and calves without underlying pathologies originated from the German Zoonoses-Monitoring program (ZoMo), the French antimicrobial surveillance program in healthy animals coordinated by the French Agency for Food, Environmental and Occupational Health & Safety (ANSES), the Norwegian monitoring program for antimicrobial resistance in bacteria from feed, food and animals (NORM-VET), and the UK AMR surveillance program coordinated by the Veterinary Medicines Directorate (VMD). Diagnostic submission isolates originated from different sample types from the German Resistance Monitoring of Veterinary Pathogens (GE*RM*-Vet) in the same animal categories (i.e., broilers, turkeys, fattening pigs, and calves) and from the French surveillance network for antimicrobial resistance in bacteria from diseased animals (RESAPATH) in broilers, turkeys, and calves. Additional phenotypic AMR data on clinical *E. coli* isolates from different sample types originated from (a) the Norwegian monitoring program for antimicrobial resistance in bacteria from feed, food and animals (NORM-VET) for broilers, turkeys, and quails between 2015 and 2018 and (b) from the AMR scanning surveillance system in veterinary pathogens from the Animal and Plant Health Agency (APHA) supported by the VMD in the United Kingdom for broilers, turkeys, cattle, and pigs between 2014 and 2017.

Data on clinical and non-clinical isolates from Germany and Norway, and data on non-clinical isolates from France and the United Kingdom, were obtained by broth microdilution according to the ISO 20776-1 [9,21,28,29,30,31], while data on clinical isolates from France and the United Kingdom were obtained by disk diffusion, using national standards [32,33].

**Figure 1:**
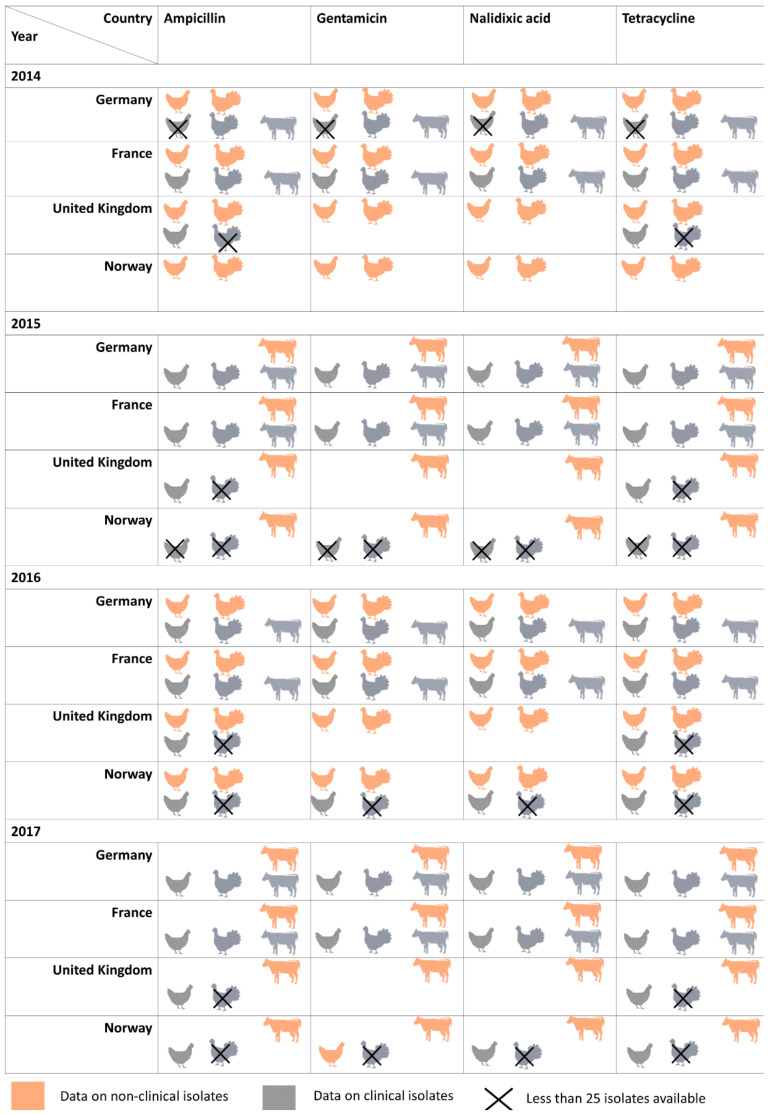
Data availability of broilers, turkeys, and calves in clinical and non-clinical isolates from 2014 to 2017 for ampicillin, gentamicin, nalidixic acid, and tetracycline across countries.

Regarding the latter databases, several limitations were encountered on overlaps of (a) antimicrobials, (b) animal categories and (c) time ranges across countries. As it can be observed in Figure 1, the largest coincidence of antimicrobials and animal categories between 2014 and 2017 was shown between Germany and France. For the UK the overlap with these antimicrobial/bacterial and animal population data was restricted to two antimicrobials (ampicillin and tetracycline) in broilers. In Norway, the number of clinical isolates that could be included was limited.

Combinations of isolate type (clinical vs. non-clinical), antimicrobial, animal category, country, and year (e.g., clinical resistance data for ampicillin in broilers from Germany in 2014) with fewer than 25 isolates were excluded. The study analyzed ampicillin, gentamicin, nalidixic acid, and tetracycline in broilers, turkeys, and calves. This reflects the overlap between animal categories and between the test panels in the monitoring systems on clinical and non-clinical isolates from Germany and France between 2014 and 2017. Although colistin was also tested in clinical and non-clinical isolates in Germany and France, it was not included in the analysis, as clinical resistance data from France are produced by disk diffusion, which is considered not reliable to test colistin susceptibility [34,35].

Only broiler data were considered from the UK and Norway due to the limited number of clinical isolates from the animal categories. UK data on clinical and non-clinical isolates were also statistically analyzed, but results were included separately because of the limited overlap on the antimicrobial panel (i.e., ampicillin and tetracycline) and animal categories (i.e., broiler). Resistance data on clinical isolates from Norway were limited, and therefore only univariable analyses were performed comparing data on clinical and non-clinical isolates from broilers in 2016.

For descriptive purposes, data on antimicrobial use per animal category from Germany, France, and the UK were assessed between 2014 and 2017. However, different AMU measurements are used. Moreover, the animal categories for which data are available differ between countries. In Germany, therapy frequency (TF) is used, expressing animal exposure to antimicrobials in days under treatment. In France, the Animal Level of Exposure to Antimicrobials (ALEA) is based on sales data. German and French data on use were assessed from the respective national reports [36,37]. UK data on usage of penicillin (amoxicillin and phenoxymethylpenicillin) and tetracycline antibiotic classes in the broiler sector originated from the British Poultry Council and were provided as grams of active ingredients by the VMD (data shown as kg of active ingredients in Appendix A). It was not possible to acquire AMU data by antimicrobial in all countries. Therefore, AMU data were collected at the antimicrobial class level providing uniformity. Due to the differences in the animal populations covered between AMU and AMR in France, AMU data were not included in the statistical models.

Additionally, an estimate of antimicrobial consumption from 2018 expressed as treated live weight per kg for broilers and turkeys was provided in France based on a sample of 10 volunteer producing organizations. Assuming that the differences in the use between broilers and turkeys remained stable over time, this difference was also considered in the discussion.

### 2.2. Overcoming the Lack of Harmonization within Countries on Antimicrobial Susceptibility Testing

Antimicrobial susceptibility tests by broth microdilution were performed according to ISO 20776-1 [9]. Data provided in this study based on broth microdilution (i.e., data on clinical and non-clinical isolates from Germany and Norway together with data on non-clinical isolates from France and the United Kingdom) were considered harmonized. Data on AST generated by disk diffusion method (i.e., data on clinical isolates from France and the United Kingdom) are based on different standards (i.e., the norm NF U 47-107 applied by the French Society of Microbiology (CASFM) and the standard of the British Society for Antimicrobial Chemotherapy (BSAC), respectively).

The NRI method assumes that data fit the normal distribution. This procedure detects the most common mode of the wild-type population (i.e., the upper part of the IZD or the lower part in a MIC distribution). Range of values covered by the normal distribution, which can be assumed to be displayed by MIC values and IZD of the wildtype isolates, are identified, calculating an objective cut-off [38]. It was applied to generate NRI cut-offs for (a) clinical isolates from Germany and Norway together with non-clinical isolates from Germany, France, the United Kingdom, and Norway; (b) clinical isolates from France; and (c) clinical isolates from the United Kingdom (Table 1). These NRI cut-offs were applied for the categorization of the AST results. NRI cut-offs were defined by the use of an Excel tool (http://www.bioscand.se/nri/ [accessed on 24 March 2021]).

Isolates with a MIC up to the MIC NRI cut-off and with an inhibition zone diameter (IZD) above the IZD NRI cut-off (i.e., wild type isolates without acquired/mutational resistance [10]) were considered microbiologically susceptible. The other isolates were considered microbiologically resistant.

### 2.3. Statistical Analysis

Data were analyzed applying “the Konstanz information Miner (KNIME)” tool (Version 4.1.2) and the software “R” (Version 3.6.3) using the CRAN packages “ROCR” and “pscl”. Several analyses were performed adopting different logistic regression approaches: (a) univariable and multivariable analyses for each antimicrobial (ampicillin, nalidixic acid, tetracycline, and gentamicin), each animal category (broilers, turkeys, and calves), and each country (Germany and France) were performed including the isolate type variable (clinical vs. non-clinical) and the year as independent variables to assess differences between clinical and non-clinical isolates. (b) Univariable analyses for each antimicrobial, animal category, country, and isolate type were performed including the year as an independent variable to assess resistance trends. The year in the model was analyzed as a numeric variable.

The separate analyses for the United Kingdom were restricted by the limited overlap of the antimicrobial panel (i.e., ampicillin and tetracycline; gentamicin and nalidixic acid were not covered) and animal categories (i.e., broiler) according to Figure 1. In Norway, univariable analyses were performed per antimicrobial for broilers in 2016 including the isolate type variable as a factor in order to assess differences between clinical and non-clinical isolates. The outcome variable (i.e., susceptible (y = 0) or resistant (y = 1)) was the qualitative categorization of (a) IZD data and (b) MIC data applying the NRI method.

Univariable and multivariable models showed significant results with a *p*-value lower than 0.05. For the explanatory factor “isolate type”, an odds ratio (OR) < 1 indicated a lower fraction of resistance in the clinical isolates compared to non-clinical isolates. For the explanatory factor “year”, an OR < 1 indicated a decrease in resistance across the years.

## 3. Results

Table 2, Table 3 and Table 4 show the resistant proportions and the number of tested isolates per year and isolate type in France and Germany for broilers, calves, and turkeys and in Norway and the UK for broilers, respectively. Univariable and multivariable logistic regression results per antimicrobial and animal type within France, Germany, the United Kingdom, and Norway are shown in Table 5 and Table 6, respectively. Table 7 shows univariable logistic regression analyses of the year per animal category, antimicrobial, and isolate type in France, Germany, and the United Kingdom.

Note that data available for the year variable differed between data on clinical (for broilers from 2015 to 2017 and for calves and turkeys from 2014 to 2017) and non-clinical isolates (for broilers and turkeys: 2014 and 2016; and for calves: 2015 and 2017) describing trends over different time frames.

### 3.1. Ampicillin

Lower resistance levels were encountered in clinical isolates compared to non-clinical isolates for broilers and turkeys in France and Germany and for broilers in the UK. In contrast, higher resistance levels in clinical isolates as compared to non-clinical isolates were found for calves in France and Germany (Table 5 and Table 6). In Norway, resistance levels in clinical and non-clinical isolates from broilers did not differ significantly in 2016 (Table 5).

Resistance levels increased over time in clinical isolates from broilers and turkeys in Germany but decreased in clinical isolates from broilers in the UK and in non-clinical isolates from calves in France (Table 7).

### 3.2. Gentamicin

Lower resistance odds in clinical isolates than in non-clinical isolates were encountered for turkeys in Germany (Table 5). Resistance levels were higher in clinical than in non-clinical isolates from broilers and calves in France and in isolates from calves in Germany (Table 5 and Table 6). In Norway, resistance levels in clinical and non-clinical isolates from broilers did not differ significantly in 2016 (Table 5).

Decreasing resistance levels across the years were encountered in clinical isolates from calves and turkeys in France and from calves in Germany (Table 7).

### 3.3. Nalidixic Acid

Analyses revealed higher resistance levels in clinical than in non-clinical isolates from calves in France and in Germany (Table 5). However, resistance levels in clinical and non-clinical isolates from broilers were similar in 2016 in Norway (Table 5).

In broilers, the model found increasing resistance to nalidixic acid in clinical isolates in France and in non-clinical isolates in Germany over time. Decreasing resistance was detected in clinical isolates from calves in France and in clinical and non-clinical isolates from turkeys in Germany (Table 7).

### 3.4. Tetracycline

Lower resistance levels were encountered in clinical than in non-clinical isolates from broilers in the UK and from broilers and turkeys in France and Germany. For calves, the analysis revealed higher resistance levels in clinical isolates than in non-clinical isolates in Germany and France (Table 5 and Table 6). In Norway, resistance levels in clinical and non-clinical isolates from broilers were similar in 2016 (Table 5).

Resistance levels to tetracycline decreased in clinical isolates from all animal categories in France across the years. In Germany, resistance levels decreased in non-clinical isolates for turkeys. In the UK, resistance levels decreased in clinical and non-clinical isolates from broilers (Table 7).

## 4. Discussion

Our hypothesis was that clinical isolates could be at higher risk of AMR than non-clinical isolates. Our study showed that this is not always the case. In Germany and France, higher resistance levels in clinical isolates were encountered to ampicillin, gentamicin, nalidixic acid, and tetracycline for calves. In broilers, this was only detected for gentamicin in France. In contrast, resistance to ampicillin and tetracycline was less likely in clinical isolates than in non-clinical isolates from broilers and turkeys in France and Germany and from broilers in the UK. This was also found for gentamicin in isolates from turkeys in Germany. Our data showed a general pattern with a higher AMR risk in clinical isolates from calves and in non-clinical isolates from broilers and turkeys, which was contrary to our hypothesis. The data analyzed had at least 41 isolates per year, isolate type, and country. The rest were discarded as they had less than 25 isolates, which is the isolates number used in a previous work as a minimum [25]. The latter threshold was based on the fact that EFSA applied a minimum number of 10 isolates to run analyses acknowledging that this number could be too low [40].

To our knowledge, this is the first study comparing resistance between clinical and non-clinical isolates of *E. coli* from different animal populations within several countries using the NRI method to harmonize results of different methods of resistance testing. A previous statistical study based on EUCAST epidemiological cut-off values with the same data on German broilers and turkeys found the same results, not confirming the hypothesis with a broader antimicrobial panel. In that previous study, additionally, lower resistance levels in clinical isolates were found to colistin for broilers and turkeys in Germany [25].

Previous studies in Estonia and Germany support our results comparing clinical and non-clinical isolates in cattle descriptively [24,41].

Resistance data from Norway did not differ significantly between clinical and non-clinical isolates to ampicillin, gentamicin, nalidixic acid, and tetracycline for broilers in 2016 (Table 2). This might be due to the limited number of clinical isolates together with the low resistance levels detected within the country.

A decreasing occurrence of resistance was found to several antimicrobials in all countries in clinical and non-clinical isolates. This indicates that effective measures are being taken in these countries to reduce resistance to antimicrobials. However, resistance increased in some animal populations to some antimicrobials.

Interestingly opposite resistance trends were observed to nalidixic acid in clinical and non-clinical isolates from broilers in countries. Resistance to nalidixic acid increased significantly in clinical isolates from broilers in France but tended to decrease in non-clinical isolates, albeit not significantly (*p* = 0.061). Conversely, in Germany, resistance to nalidixic acid decreased significantly in non-clinical isolates and tended to increase in clinical isolates from broilers in Germany, albeit not significantly (*p* = 0.086). A plausible reason for these results might be that specific strain types are associated with *E. coli* pathogens that are tested because they caused disease [42,43], while non-clinical *E. coli* isolates are randomly selected isolates, and therefore the selection basis differs [44].

Some resistance differences were found between countries. In France, resistance to tetracycline tended to be higher than in Germany in isolates from all animal categories and to ampicillin in isolates from calves. In contrast, German isolates showed higher resistance levels to nalidixic acid in isolates from all animal categories. These differences might be partially explained by differences in AMU patterns.

In France, the highest occurrence of resistance in isolates was encountered to ampicillin and tetracycline and the lowest to nalidixic acid and gentamicin for all three animal categories. This is in line with the highest level of use in poultry and cattle for penicillins and tetracyclines and the lowest for fluoroquinolones and aminoglycosides. The stratified use data for 2018 [45] indicate that the predominance of penicillins and tetracyclines was observed in both poultry species.

In Germany, differences in resistance levels were observed between the animal species. Resistance to ampicillin, tetracycline, and fluoroquinolones was high in all three animal populations. Resistance to gentamicin was only high in clinical isolates from calves. The highest occurrence of resistance in isolates from calves was found for tetracycline and ampicillin and lowest for nalidixic acid and gentamicin. This was in line with the use data. Resistance association with use was already described for broilers and turkeys [25].

In the UK, higher resistance proportions in isolates from broilers were found to ampicillin than to tetracycline, this being in line with the use data.

In Norway, the highest resistance prevalence in isolates was found for ampicillin and nalidixic acid and lower for tetracycline and gentamicin for broilers. These resistance proportions in isolates were considerably low and were in line with null or very low AMU.

In France, exposure to penicillins was higher in poultry than in cattle, while resistance was the highest in isolates from calves and the lowest in those from broilers [32]. We observed that AMU for poultry tended to decrease from 2014 to 2017 for the studied antimicrobial classes. According to the French estimate for 2018 [45], the use of penicillins was higher in turkeys than in broilers, which would be in line with the different occurrence of resistance to ampicillin. In Germany, the use of penicillins was by far the highest in turkeys. However, like in France, the highest resistance proportions to ampicillin were detected in isolates from calves, while those from broilers and turkeys were similar.

The use of penicillins in broilers in the UK decreased drastically across the years. That is in line with the decrease in resistance to ampicillin in clinical isolates from broilers. However, no decrease was observed in the non-clinical isolates.

The association between the use of penicillins and resistance to ampicillin is confirmed by data from countries with a very low total of AMU in mg/PCU from 2014 to 2017 such as Sweden or Norway that showed resistance levels low or relatively low to ampicillin (<26%) in non-clinical isolates from broilers, turkeys, and calves [10,46,47,48]. This study shows that these low values are also reported for the clinical isolates from broilers in Norway.

The high resistance rates to ampicillin for calves in France and Germany may also partially be attributed to feeding waste milk from dairy cows to the calves, which has been shown to influence AMR in calves [49]. Furthermore, the type of penicillin used for treatment may be a relevant factor for the resistance development. While aminopenicillin usage has been correlated strongly with resistance to ampicillin, no association has been found between penicillin usage and ampicillin resistance in several countries [50]. Aminopenicillins affect Gram-positive and Gram-negative bacteria, promoting further resistance. Natural penicillins and related compounds [51] only work against Gram-positives and thus do not directly select for resistance in Gram-negatives.

With the exception of German broilers, the use of aminoglycosides was very low in France and Germany [32,36]. Similar low resistance prevalence was found in both countries and in Norway in non-clinical isolates for the three animal categories. Clinical isolates from calves had substantially higher resistance rates than isolates from the other categories. This higher level of resistance might not be attributed to differences in use, as this difference was small in France and in Germany. The highest use by far was observed in broilers. According to the stratified use data for 2018 [45], aminoglycoside use in broilers and turkeys was similar and very low in France. Data on AMU and AMR in France refer to different animal categories (cattle vs. calf), i.e., it is not clear, which share of the antimicrobials used in the summary category cattle are used in calves.

Resistance to gentamicin is not frequent in most animal bacteria, while resistance to streptomycin and spectinomycin is high in animal pathogens [52]. The type of aminoglycoside applied for the treatment might be a key factor in the resistance development as it has been reported for penicillins.

In Norway, the use of aminoglycosides in broilers and turkeys was negligible or non-existent. Accordingly, resistance proportions in isolates from broilers were very low in clinical and non-clinical isolates.

Fluoroquinolones are highest priority critically important antimicrobials for humans. The association between the use of fluoroquinolones and their related resistance in isolates from livestock is shown by the lower occurrence of resistance to fluoroquinolones in isolates from countries where these drugs are not licensed for use in animals or specific animal populations [40,53]. Fluoroquinolone exposure in France and Germany was higher in poultry than in cattle from France [32] and in calves from Germany [36], which is mirrored by resistance to nalidixic acid in non-clinical isolates. Higher fluoroquinolone use was observed in broilers than in turkeys in the French estimate of 2018 [45], and accordingly, resistance to nalidixic acid was higher in isolates from broilers than in those from turkeys.

In Germany, TF of calves with fluoroquinolones was the lowest. TF of broilers was 3 times higher and TF of turkeys 5 to 7 times higher. The highest resistance rates to nalidixic acid were seen in non-clinical isolates from broilers, followed by turkeys and then calves. Fluoroquinolone use for broilers and calves was in line with resistance to nalidixic acid. However, resistance to nalidixic acid in turkeys was not. The presence of quinolone resistance genes showing little or no fitness cost, such as *gyrA* [54], could make it difficult to demonstrate the relationship between the use and resistance, as non-use will not necessarily lead to a decrease in resistance.

In Norway, the use of fluoroquinolones in broilers and turkeys was null. Accordingly, resistance proportions in isolates from broilers were very low in clinical and non-clinical isolates.

Tetracyclines are frequently applied as livestock treatments representing about 28.0%, 40.5%, and 60.9% of all sold veterinary drugs in 2014 and about 26.3%, 39.3%, and 41.2% in 2017 in Germany, France, and the United Kingdom, respectively [20]. This high selective pressure for tetracycline in livestock can explain the high occurrence of resistance in clinical and non-clinical isolates of *E. coli*.

In Germany, the highest resistance to tetracycline was detected in isolates from calves and lowest from broilers, which was in line with the differences in use levels.

The use of tetracyclines in France was higher in poultry than in cattle. However, tetracycline use was higher in turkeys than in broilers according to the estimate for 2018 [45]. This agrees with resistance to tetracycline in isolates. Resistance prevalence was higher in isolates from turkeys and calves than in those from broilers.

In Norway, the use of tetracyclines in broilers and turkeys was negligible or non-existent. Accordingly, resistance proportions in isolates from broilers were very low in clinical and non-clinical isolates.

The use of tetracycline in broilers in the UK decreased drastically across the years, which is in line with decreasing resistance proportions of clinical and non-clinical isolates from broilers.

In addition to all the factors mentioned above, the presence of multi-resistant bacteria and the phenomenon of co-resistance might also explain the instances of non-agreement in trends of AMU and AMR [55,56].

Data on non-clinical isolates are routinely collected for some animal populations and food items by EFSA at the EU level, while data on clinical isolates are not commonly and routinely collected and reported to the EU-level by most EU countries. In line with previous reports [24,25], we found different occurrence of resistance in clinical and non-clinical isolates, although both are at the national population level exposed to the same level of antimicrobial use. One reasonable explanation for the differences observed between clinical and non-clinical isolates might be the different selection procedure for the strains. Specific *E. coli* strains prevail in the isolates from diagnostic submissions because of their pathogenicity [25]. In contrast, commensal *E. coli* are selected randomly from a broad range of available subtypes of *E. coli* that typically colonize the mammalian and avian gut. A further plausible reason might be that clinical samples are not randomly collected from all farms, and that farms submitting such samples may differ from other farms. However, this does not seem to have the same effect in the different animal populations studied. We found in most cases higher resistance risk in clinical isolates for calves and lower for broilers and turkeys within each country.

There are some arguments to be cautious of when interpreting differences between isolates in results. Caveats were detected comparing data on clinical and non-clinical isolates in Germany and France for broilers, turkeys, and calves: (a) type of data collection basis (i.e., mandatory; non-clinical isolates vs. voluntary; clinical isolates); (b) sample collection at the slaughterhouse, i.e., at the end of the production period vs. during the lifetime or post mortem; (c) samples from caeca (non-clinical) vs. samples from different tissues and materials (clinical); (d) data representative for the animal population in the country vs. data representative for the samples examined in the laboratories contributing to the system; (e) maximum of one sample per flock/herd and year (non-clinical) vs. possibility of more than one sample per flock/herd and year (clinical); (f) data availability in non-clinical isolates (2014 and 2016 for broilers and turkeys; 2015 and 2017 for calves) vs. in clinical isolates (from 2014 to 2017); and (g) data analyzed in one laboratory (i.e., French and UK data on non-clinical isolates together with Norwegian and German data on clinical and non-clinical isolates) vs. several laboratories (i.e., French data on clinical isolates).

Further studies with more explanatory variables such as farm management (e.g., organic vs. conventional, farm-level antimicrobial use), resistance levels of freshly hatched birds, molecular typing, genomic data, a common suitable AMU unit that represents each animal category (i.e., broilers, turkeys and calves), and longer periods are required (1) to better assess the association between AMU and AMR across and within countries and (2) to clarify the differences found between clinical and non-clinical isolates.

This study does not define the pathogenicity of the isolates. It is assumed that many of the clinical isolates are pathogenic *E. coli*. However, the resistance of these isolates to antimicrobials has only been investigated phenotypically. Similarly, *E. coli* isolates from healthy animals are assumed to be non-pathogenic, but they might be pathogenic under specific cases.

This study underlines the need for harmonization of AMU and AMR monitoring and surveillance systems within and between countries [16] in order to continue advancing in the understanding of the AMR development and the association between AMU and AMR within and between sectors in a One Health approach. A common set of antimicrobials investigated across countries, as has been achieved for the non-clinical isolates, should also be targeted for the clinical isolates.

Associations between AMU and AMR also need to be interpreted with care. Use data are at the country level rather than regional or farm level and represent different animal categories than AMR data. Use data in France are available on cattle and poultry, while resistance data are on calves (i.e., a subgroup of cattle), broilers, and turkeys. Large differences have been identified in the use of antimicrobials between broilers and turkeys in Germany [36]. In France, separate data on AMU in broilers and turkeys were only available from an estimate in 2018 from a sample of volunteer producing organizations. They are not necessarily representative of the whole use for broiler and turkey populations in France. This suggests that AMU collected by type of production (e.g., broiler or laying hens instead of chicken), animal categories (e.g., broilers and turkeys instead of poultry), and age categories when the animal production cycle is long (e.g., calves instead of cattle) might provide a more suitable and accurate data basis to assess the association between AMU and AMR.

Direct comparisons of susceptible and resistant isolates based on different standards without using any statistical method to overcome the lack of harmonization of AMR on laboratory methodologies might be possible if the same CBPs were accidentally shown. In this study, the NRI method was applied as a straightforward approach to interpret AST results. NRI cut-offs for MIC values were calculated by using all broth microdilution data from France, Germany, the United Kingdom, and Norway for *E. coli*. Resulting NRI cut-offs and published EUCAST ECOFFs were similar. According to the results, identical cut-offs were obtained for gentamicin (2 mg/L) and nalidixic acid (8 mg/L). A difference of one dilution step in the cut-offs was estimated for ampicillin (NRI = 16 mg/L; ECOFF = 8 mg/L) and tetracycline (NRI = 4 mg/L; ECOFF = 8 mg/L). No significant differences were detected applying NRI cut-offs and ECOFFs in results (data not shown). These results together with the fact that the NRI method is an objective approach for the estimation of the wild-type populations in MIC and IZ distributions [27] encouraged us to use this method.

Our approach to calculating NRI cut-offs tried to make the best use of available data. EUCAST has a defined SOP for doing these calculations, but with the available data we could only do the calculations violating this SOP, with respect to the number of laboratories and isolates to be included [57]. Therefore, our NRI cut-offs cannot claim to be fully accurate. However, doing the calculations on different sub-sets of the data produced very similar results, which encouraged us to proceed.

Our study showed that the NRI method can be used to set a harmonized interpretative criterion in order to compare non-harmonized resistance data, provided that quantitative data across a substantial range of values are available. Therefore, the NRI method might be regularly used in veterinary medicine and in One Health studies until international harmonization of AST is achieved [26,27]. We concede that this issue might also be addressed by applying other statistical methods proposed, and further research comparing the approaches is warranted [13,58,59].

The lack of harmonization on AMU has not yet been overcome. Differences in units and in populations covered prevented the inclusion of AMU in the statistical models. Much can be gained if the requirement of Reg (EU) No. 6/2019 to collect consumption data is utilized to improve the comparability of data and populations in this area.

## 5. Conclusions

The Normalized Resistance Interpretation (NRI) approach, a method to identify the wild-type distribution, provides approximate epidemiological cut-offs, provided that quantitative data across a substantial range of values are available. These NRI cut-offs allow comparing quantitative results from antimicrobial resistance (AMR) systems with different levels of harmonization regarding the laboratory methods and procedures. This allowed comparing clinical and non-clinical isolates from animal categories in countries. Until AMR systems are globally harmonized, the NRI method may be considered an alternative to define interpretative values in order to compare and overcome lack of harmonization issues on AMR monitoring and surveillance systems. This method could be applied to mitigate AMR, advising political decisions.

In line with our hypothesis, a higher resistance risk was found in clinical than in non-clinical isolates from calves to all four included antimicrobials (i.e., ampicillin, gentamicin, nalidixic acid, and tetracycline) in Germany and France. In isolates from poultry, this was only found for gentamicin in broilers in France. In contrast to our hypothesis, a higher probability of resistance in non-clinical isolates was encountered for ampicillin and tetracycline for broilers in France, Germany, and the UK; for turkeys in France and Germany; and to gentamicin for turkeys in Germany. This suggests that the higher presence of resistance in one isolate type than the other (i.e., clinical or non-clinical isolates) is associated with the relationship between animal categories and the antimicrobial that might be related to how animals are treated following disease. This could also be attributable to other reasons such as co-selection or expansion of a successful clone. Resistance prevalence did not differ between clinical and non-clinical isolates from broilers in Norway in 2016. This might be due to the low number of isolates together with the low resistance prevalence in Norway for broilers.

Differences between countries were observed for specific isolate type–drug–population combinations on a descriptive level. These findings were mostly in line with differences in antimicrobial use (AMU), although comparison of these data has many caveats due to differences in sampling, reporting, in units of measurement, and granularity of available data.

Associations between AMU per antimicrobial class and AMR have been described in this work. However, data on AMU per drug for the same animal type as data on AMR may provide further information that ease identification of associations between AMU and AMR.

The analysis showed in most cases decreasing resistance trends in clinical and/or non-clinical isolates over time for the antimicrobials and animal categories studied in Germany, France, and the United Kingdom, suggesting that measures carried out against AMR including the reduction in AMU in each country have effective results.

## 6. Patents

The NRI method was used with permission from the patent holder, Bioscand AB, TÄBY, Sweden (European patent No 1383913, US Patent No. 7,465,559). The automatic and manual excel programs were made available through courtesy of P. Smith, W. Finnegan, and G. Kronvall.

## Figures and Tables

**Table 1:** NRI cut-offs calculated and the corresponding isolates used for the determination together with broth microdilution ECOFFs from EUCAST (29 June 2020).

Antimicrobial Drug Tested	Cut-Offs (Number of Isolates Tested for the Determination)			
	Ampicillin	Nalidixic Acid	Tetracycline	Gentamicin
Epidemiological cut-off values (ECOFFs-EUCAST) for broth microdilution (mg/L)	>8 (73,390)	>8 (39,317)	>8 (17,276)	>2 (80,274)
French NRI cut-offs adopting all IZD data (mm)	<17 (5792)	<22 (39,317)	<20 (51,882)	<20 (55,901)
NRI cut-offs adopting all IZD data (mm) from the United Kingdom	<11 (2793)		<19 (2684)	
NRI cut-offs adopting all broth microdilution data from France, Germany, the United Kingdom and Norway (mg/L)	>16 (8381)	>8 (8379)	>4 (8373)	>2 (8372)

IZD: inhibition zone diameter; NRI: normalized resistance interpretation.

**Table 2:** Resistant proportions applying the corresponding NRI cut-offs and numbers of clinical and non-clinical *Escherichia coli* isolates in brackets reported for broilers in Norway, the United Kingdom, France, and Germany between 2014 and 2017.

Country Drug	2014		2015	2016		2017
	Clinical	Non-Clinical ^2^	Clinical	Clinical	Non-Clinical ^2^	Clinical
Norway						
AMP	na	6.3 (205)	6.3 (16) ^1^	2.3 (43)	3.9 (181)	10.4 (77)
GEN	na	0.0 (205)	0.0 (16) ^1^	2.3 (43)	0.6 (181)	3.9 (77)
NAL	na	3.4 (205)	0.0 (16) ^1^	4.7 (43)	6.1 (181)	13.0 (77)
TET	na	1.5 (205)	6.3 (16) ^1^	4.7 (43)	3.3 (181)	16.9 (77)
UK						
AMP	52.4 (103)	73.6 (159)	56.1 (171)	34.7 (170)	79.5 (303)	26.7 (75)
TET	47.6 (103)	60.4 (159)	53.2 (171)	36.5 (170)	50.5 (303)	25.3 (75)
France						
AMP	28.7 (411)	55.9 (227)	32.4 (519)	29.1 (515)	55.9 (188)	27.8 (421)
GEN	5.5 (1352)	1.8 (227)	6.2 (2406)	5.5 (3357)	3.2 (188)	5.1 (4156)
NAL	34.8 (881)	43.7 (227)	42.1 (1963)	47.5 (2878)	34.7 (188)	45.9 (3650)
TET	49.8 (1495)	63.4 (227)	45.8 (2638)	44.6 (3164)	62.2 (188)	45.8 (3453)
Germany						
AMP	50.0 (18) ^1^	55.2 (230)	30.3 (76)	32.0 (50)	59.3 (177)	56.1 (41)
GEN	5.5 (18) ^1^	7.0 (230)	2.7 (75)	12.0 (50)	6.8 (177)	7.3 (41)
NAL	44.4 (18) ^1^	44.8 (230)	67.1 (76)	58.0 (50)	56.5 (177)	46.3 (41)
TET	44.4 (18) ^1^	33.5 (230)	17.3 (75)	14.0 (50)	28.8 (177)	31.7 (41)

^1^ Not included in the analysis as less than 25 isolates were tested; ^2^ Data on non-clinical isolates are collected every two years according to the EU-legislation [9,39]; AMP: ampicillin; GEN: gentamicin; NAL: nalidixic acid; TET: tetracycline.

**Table 3:** Resistant proportions applying the corresponding NRI cut-offs and numbers of clinical and non-clinical *Escherichia coli* isolates reported for calves in France and Germany between 2014 and 2017.

Country Drug	2014	2015		2016	2017	
	Clinical	Clinical	Non-Clinical ^1^	Clinical	Clinical	Non-Clinical ^1^
France						
AMP	79.3 (527)	84.1 (592)	53.5 (202)	83.6 (477)	83.9 (342)	45.0 (202)
GEN	23.3 (2668)	21.6 (3814)	5.9 (202)	20.9 (4543)	20.2 (4117)	4.4 (202)
NAL	47.1 (1124)	42.8 (2203)	12.4 (202)	(41.5 (2859)	35.5 (2454)	9.4 (202)
TET	79.9 (2290)	78.0 (3542)	72.8 (202)	76.2 (4323)	76.4 (3900)	65.8 (202)
Germany						
AMP	70.9 (206)	70.5 (207)	31.8 (192)	65.3 (121)	75.0 (112)	35.5 (242)
GEN	37.9 (203)	29.9 (204)	0.5 (192)	20.7 (121)	25.9 (112)	3.3 (242)
NAL	50.5 (206)	56.6 (205)	10.4 (192)	48.8 (121)	50.9 (112)	8.7 (242)
TET	63.7 (204)	63.2 (204)	38.5 (192)	58.7 (121)	67.9 (112)	38.0 (242)

^1^ Data on non-clinical isolates are collected every two years according to the EU-legislation [9,39]; AMP: ampicillin; GEN: gentamicin; NAL: nalidixic acid; TET: tetracycline.

**Table 4:** Resistant proportions applying the corresponding NRI cut-offs and numbers of tested clinical and non-clinical *Escherichia coli* isolates reported for turkeys in France and Germany between 2014 and 2017.

Country Drug	2014		2015	2016		2017
	Clinical	Non-Clinical ^1^	Clinical	Clinical	Non-Clinical ^1^	Clinical
France						
AMP	41.6 (113)	64.4 (239)	34.8 (135)	45.9 (109)	67.0 (182)	46.2 (117)
GEN	3.8 (640)	4.2 (239)	4.3 (1188)	2.1 (1478)	1.1 (182)	1.7 (1552)
NAL	20.0 (551)	20.9 (239)	19.8 (1097)	22.2 (1426)	23.1 (182)	20.3 (1401)
TET	48.0 (783)	75.3 (239)	47.3 (1401)	41.8 (1345)	67.6 (182)	38.7 (1219)
Germany						
AMP	37.8 (82)	64.1 (184)	36.5 (104)	37.9 (95)	63.3 (188)	57.1 (63)
GEN	2.5 (80)	10.3 (184)	3.8 (104)	3.2 (95)	6.4 (188)	9.5(63)
NAL	49.4 (81)	32.6 (184)	22.9 (105)	24.2 (95)	22.3 (188)	20.6 (63)
TET	41.3 (80)	56.0 (184)	22.1 (104)	17.9 (95)	43.6 (188)	30.2 (63)

^1^ Data on non-clinical isolates are collected every two years according to the EU-legislation [9,39]; AMP: ampicillin; GEN: gentamicin; NAL: nalidixic acid; TET: tetracycline.

**Table 5:** Univariable logistic regression analyses per animal category and antimicrobial in France, Germany, the United Kingdom, and Norway.

Animal Category Drug	Factor	France		Germany		UK		Norway ^1^	
		*p*-Value	OR (95% CI)	*p*-Value	OR (95% CI)	*p*-Value	OR (95% CI)	*p*-Value	OR (95% CI)
Broilers									
AMP	Isolate type	<0.001	0.34 (0.27–0.42)	<0.001	0.45 (0.31–0.65)	<0.001	0.23 (0.17–0.3)	0.628	0.59 (0.03–3.45)
	Year	<0.001	0.9 (0.83–0.97)	0.97	1.0 (0.86–1.17)	0.001	0.8 (0.7–0.92)	na	na
GEN	Isolate type	0.008	2.37 (1.33–4.77)	0.913	0.97 (0.45–1.93)	na	na	0.307	4.28 (0.16–109.86)
	Year	0.456	0.98 (0.91–1.05)	0.673	1.07 (0.78–1.47)	na	na	na	na
NAL	Isolate type	0.043	1.24 (1.01–1.51)	0.0529	1.44 (1.0–2.08)	na	na	0.72	0.75 (0.11–2.95)
	Year	<0.001	1.12 (1.08–1.17)	0.034	1.19 (1.02–1.4)	na	na	na	na
TET	Isolate type	<0.001	0.51 (0.41–0.62)	0.006	0.55 (0.35–0.83)	<0.001	0.63 (0.49–0.81)	0.673	1.42 (0.20–6.43)
	Year	0.002	0.95 (0.92–0.98)	0.172	0.89 (0.74–1.06)	<0.001	0.75 (0.65–0.85)	na	na
Calves									
AMP	Isolate type	<0.001	4.92 (3.92–6.18)	<0.001	4.66 (3.59–6.06)	na	na	na	na
	Year	0.005	0.89 (0.81–0.97)	<0.001	0.81 (0.73–0.9)	na	na	na	na
GEN	Isolate type	<0.001	4.95 (3.27–7.93)	<0.001	20.24 (10.85–43.09)	na	na	na	na
	Year	<0.001	0.94 (0.91–0.98)	<0.001	0.62 (0.53–0.72)	na	na	na	na
NAL	Isolate type	<0.001	5.65 (4.17–7.85)	<0.001	10.14 (7.18–14.67)	na	na	na	na
	Year	<0.001	0.86 (0.82–0.89)	<0.001	0.71 (0.63–0.8)	na	na	na	na
TET	Isolate type	<0.001	1.51 (1.22–1.87)	<0.001	2.79 (2.18–3.6)	na	na	na	na
	Year	<0.001	0.94 (0.9–0.97)	0.003	0.85 (0.77–0.95)	na	na	na	na
Turkeys									
AMP	Isolate type	<0.001	0.38 (0.29–0.5)	<0.001	0.4 (0.3–0.54)	na	na	na	na
	Year	0.441	0.96 (0.85–1.08)	0.857	1.02 (0.88–1.17)	na	na	na	na
GEN	Isolate type	0.892	0.96 (0.55–1.85)	0.035	0.51 (0.27–0.94)	na	na	na	na
	Year	<0.001	0.72 (0.62–0.84)	0.624	0.94 (0.7–1.25)	na	na	na	na
NAL	Isolate type	0.598	0.94 (0.74–1.2)	0.628	0.93 (0.68–1.27)	na	na	na	na
	Year	0.635	1.02 (0.96–1.09)	<0.001	0.76 (0.65–0.88)	na	na	na	na
TET	Isolate type	<0.001	0.31 (0.25–0.38)	<0.001	0.38 (0.28–0.51)	na	na	na	na
	Year	<0.001	0.83 (0.78–0.87)	<0.001	0.74 (0.64–0.86)	na	na	na	na

^1^ Univariable analyses for Norway were performed per antimicrobial for broilers only in 2016 including only the isolate type variable as a factor; AMP: ampicillin; GEN: gentamicin; NAL: nalidixic acid; TET: tetracycline.

**Table 6:** Multivariable logistic regression analyses per animal category and antimicrobial in France, Germany, and the United Kingdom.

Animal Category Drug	Factor	France		Germany		UK	
		*p*-Value	OR (95% CI)	*p*-Value	OR (95% CI)	*p*-Value	OR (95% CI)
Broilers							
AMP	Isolate type	<0.001	0.34 (0.27–0.43)			<0.001	0.23 (0.17–0.3)
	Year	0.533	0.98 (0.9–1.06)			0.005	0.81 (0.7–0.94)
NAL	Isolate type	0.382	1.1 (0.9–1.35)	0.236	1.28 (0.86–1.91)	na	na
	Year	<0.001	1.12 (1.07–1.16)	0.142	1.15 (0.96–1.36)	na	na
TET	Isolate type	<0.001	0.52 (0.43–0.64)			<0.001	0.64 (0.5–0.83)
	Year	0.037	0.97 (0.93–1.0)			<0.001	0.75 (0.66–0.86)
Calves							
AMP	Isolate type	<0.001	5.03 (3.97–6.39)	<0.001	4.85 (3.66–6.47)	na	na
	Year	0.492	1.04 (0.94–1.15)	0.481	1.05 (0.93–1.19)	na	na
GEN	Isolate type	<0.001	4.86 (3.21–7.78)	<0.001	17.23 (9.11–37.02)	na	na
	Year	0.002	0.95 (0.91–0.98)	0.017	0.83 (0.71–0.97)	na	na
NAL	Isolate type	<0.001	5.49 (4.05–7.64)	<0.001	9.82 (6.82–14.45)	na	na
	Year	<0.001	0.87 (0.83–0.9)	0.584	0.97 (0.85–1.1)	na	na
TET	Isolate type	<0.001	1.48 (1.19–1.83)	<0.001	2.82 (2.15–3.71)	na	na
	Year	<0.001	0.94 (0.9–0.97)	0.877	1.01 (0.9–1.14)	na	na
Turkeys							
TET	Isolate type	<0.001	0.34 (0.27–0.42)	<0.001	0.41 (0.3–0.56)	na	na
	Year	<0.001	0.87 (0.82–0.92)	0.002	0.79 (0.68–0.92)	na	na

AMP: ampicillin; GEN: gentamicin; NAL: nalidixic acid; TET: tetracycline.

**Table 7:** Univariable logistic regression analyses of the year per animal category, antimicrobial, and isolates type in France, Germany, and the United Kingdom.

Animal Category Drug	Factor	France		Germany		UK	
		*p*-Value	OR (95% CI)	*p*-Value	OR (95% CI)	*p*-Value	OR (95% CI)
Broilers							
AMP	Clinical isolates	0.496	0.97 (0.89–1.07)	0.011	1.67 (1.13–2.49)	<0.001	0.64 (0.53–0.77)
	Non-clinical isolates	0.984	1.0 (0.83–1.22)	0.407	1.09 (0.9–1.33)	0.148	1.18 (0.94–1.48)
GEN	Clinical isolates	0.166	0.95 (0.88–1.03)	0.218	1.6 (0.76–3.46)	na	na
	Non-clinical isolates	0.351	1.36 (0.72–2.7)	0.944	0.99 (0.67–1.46)	na	na
NAL	Clinical isolates	<0.001	1.13 (1.09–1.18)	0.086	0.72 (0.49–1.05)	na	na
	Non-clinical isolates	0.061	0.83 (0.68–1.01)	0.011	1.3 (1.06–1.58)	na	na
TET	Clinical isolates	0.038	0.97 (0.93–1.0)	0.108	1.47 (0.92–2.35)	<0.001	0.7 (0.58–0.84)
	Non-clinical isolates	0.801	0.98 (0.8–1.2)	0.315	0.9 (0.73–1.11)	0.043	0.82 (0.67–0.99)
Calves							
AMP	Clinical isolates	0.079	1.11 (0.99–1.24)	0.804	1.03 (0.88–1.2)	na	na
	Non-clinical isolates	0.091	0.85 (0.7–1.03)	0.410	1.09 (0.9–1.34)	na	na
GEN	Clinical isolates	0.002	0.95 (0.92–0.98)	0.003	0.79 (0.67–0.93)	na	na
	Non-clinical isolates	0.503	0.86 (0.55–1.34)	0.078	2.56 (1.09–11.04)	na	na
NAL	Clinical isolates	<0.001	0.87 (0.83–0.9)	0.745	0.98 (0.85–1.13)	na	na
	Non-clinical isolates	0.339	0.86 (0.63–1.18)	0.539	0.91 (0.66–1.26)	na	na
TET	Clinical isolates	<0.001	0.94 (0.91–0.98)	0.778	1.03 (0.89–1.19)	na	na
	Non-clinical isolates	0.132	0.85 (0.69–1.05)	0.911	0.99 (0.82–1.21)	na	na
Turkeys							
AMP	Clinical isolates	0.209	1.12 (0.95–1.32)	0.036	1.26 (1.02–1.55)	na	na
	Non-clinical isolates	0.578	1.06 (0.87–1.3)	0.867	0.99 (0.8–1.22)	na	na
GEN	Clinical isolates	<0.001	0.72 (0.61–0.85)	0.089	1.57 (0.95–2.7)	na	na
	Non-clinical isolates	0.08	0.51 (0.2–1.0)	0.173	0.77 (0.53–1.12)	na	na
NAL	Clinical isolates	0.618	1.02 (0.95–1.1)	0.003	0.71 (0.57–0.89)	na	na
	Non-clinical isolates	0.596	1.07 (0.85–1.35)	0.033	0.79 (0.64–0.99)	na	na
TET	Clinical isolates	<0.001	0.87 (0.82–0.92)	0.062	0.81 (0.64–1.01)	na	na
	Non-clinical isolates	0.081	0.83 (0.67–1.03)	0.017	0.78 (0.64–0.96)	na	na

AMP: ampicillin; GEN: gentamicin; NAL: nalidixic acid; TET: tetracycline.

## Data Availability

The data presented in this study are openly available in the Zenodo platform (https://zenodo.org/record/4581137 [accessed on 24 March 2021]).

## Appendix A.

**Table A1:** Penicillins and tetracyclines antibiotic class usage for broilers in the UK expressed as kg of active ingredient from 2014 to 2017.

Drug\Year	2014	2015	2016	2017
Penicillins *	16,122.4	11,209.2	8879.5	6612.2
Tetracyclines	12,446.0	6655.7	3310.4	712.0

* Penicillins = amoxicillin and phenoxymethylpenicillin.
